# The Immunomodulatory Functions of Mesenchymal Stromal/Stem Cells Mediated via Paracrine Activity

**DOI:** 10.3390/jcm8071025

**Published:** 2019-07-12

**Authors:** Yueyuan Zhou, Yusuke Yamamoto, Zhongdang Xiao, Takahiro Ochiya

**Affiliations:** 1 State Key Laboratory of Bioelectronics, School of Biological Science and Medical Engineering, Southeast University, Nanjing 210096, China; 2 Division of Cellular Signaling, National Cancer Center Research Institute, 5-1-1 Tsukiji, Chuo-ku, Tokyo 104-0045, Japan; 3 Department of Molecular and Cellular Medicine, Institute of Medical Science, Tokyo Medical University, 6-1-1 Shinjuku, Shinjuku-ku, Tokyo 160-8402, Japan

**Keywords:** MSC, immune regulation, paracrine mechanism, immune diseases

## Abstract

Mesenchymal stromal/stem cells (MSCs) exist in almost all tissues, possessing the potential to differentiate into specialized cell types and exert immunomodulatory functions. Thus, they have attracted much attention as a promising therapeutic candidate. Recent studies have demonstrated that paracrine signaling is mainly responsible for the involvement of MSCs in the modulation of immune responses and the progression of diseases. Through release of secretome consisting of a diverse range of cytokines, chemokines, and extracellular vesicles (EVs), MSCs convey regulatory messages to recipient immune cells in the microenvironment. In this review, we focus on the recent advances in how MSCs contribute to immunomodulation through the secretion of paracrine factors. The further improved understanding of the molecular mechanism underlying the interactions between MSCs and immune cells highlights the paracrine biology of MSCs in the modulation of the immune microenvironment and promotes the clinical application of MSCs in regenerative medicine and immune diseases.

## 1. Introduction

Mesenchymal stromal/stem cells (MSCs) were initially identified based on their clonogenic capability in guinea-pig bone marrow and called colony-forming unit-fibroblasts (CFU-F) [1]. Subsequently, morphologically similar fibroblast-like cells were readily isolated from both fetal and adult sources, such as the umbilical cord, adipose tissue, skin, dental pulp and liver [2,3,4,5]. These cells have a specific surface-molecule phenotype, being positive for CD105, CD73, and CD29 expression and negative for CD31, CD34, CD45, CD14 and human leukocyte antigen (HLA)-DR expression, according to the criteria proposed by the International Society for Cellular Therapy (ISCT) [6]. MSCs were further shown to possess self-renewing potential and to differentiate into multiple mesodermal cell lineages under specific experimental and physiological conditions, which made them an alternative source in tissue repair and regenerative medicine [7,8,9]. In addition to transdifferentiation, the paracrine effects of MSCs are frequently correlated with the therapeutic benefits of these cells [10,11,12]. MSCs contribute to cell migration/stimulation, angiogenesis, and antiapoptotic processes through releasing various types of secretome. In particular, it was recently demonstrated that MSCs play a critical role in regulating the inflammatory microenvironment and interacting with immune cells, including T cells, B cells, natural killer (NK) cells, and dendritic cells (DCs) [13,14]. The cross talk and interplay of MSCs and local environment reversely control and regulate the paracrine activity of MSCs [15,16]. Therefore, the paracrine potency might vary with sources and microenvironment of MSCs. MSCs isolated from fetal tissues such as umbilical cord (UC) and UC-blood (UCB) were shown to have increased secretion of proinflammatory proteins and growth factors than MSCs obtained from adult adipose tissue or bone marrow [17,18]. Despite the transplantation of the same human UCB-derived MSCs (UCB-MSCs), the protective benefits are associated with significant upregulation of vascular endothelial growth factor (VEGF) and hepatocyte growth factor against hyperoxic conditions in neonatal lung injury model [19]. Due to their immunomodulatory properties, MSCs hold beneficial promise in the treatment of allograft rejection episodes, as well as the suppression of abnormal immune responses in autoimmune and inflammatory diseases. MSCs have emerged as a more appropriate option for cell therapy because of their easier isolation procedures, great expansion ability and biosafety profile, and lower ethical challenges, as well as lower risk of tumorgenicity compared to other cell sources [20,21]. Preclinical animal studies of MSC therapy have been conducted in organ transplantation, graft-versus-host disease (GVHD), multiple sclerosis, hepatic failure, lung injury, diabetes and rheumatoid arthritis [22,23,24,25,26,27,28]. The preclinical data raise the notable expectation of the application of MSCs in human projects; however, the clinical outcomes of advanced trials fell short of expectations compared to the outcomes in animal models due to many challenges that still remain to be overcome prior to the efficient clinical application of MSC-based therapy. Several issues, including the suitable source, the well-characterized population, and the clearly-determined functions of MSCs, are critical to achieve the appropriate therapeutic effects. The paracrine products of MSCs are considered as the alternative to cell-based therapy as cell-free therapy. The secretome of MSCs differs depending on the tissue from which the MSCs are obtained, and substantial variation between donors and in response to different culture conditions [29,30]. Although there a number of reports of improved outcomes from the clinical application of MSCs, the evidence to date has not supported the conclusion that they are effective therapy. Therefore, it is critical to explore the in-depth mechanisms of MSCs involved in the immune system, especially their paracrine biology, including the regulation and mechanism of secretion of soluble factors and extracellular vesicles (EVs).

## 2. Paracrine Hypothesis of MSCs

Intramyocardial injection of stem cells has been extensively proven to offer therapeutic benefits in infarct repair by promoting myocardium regeneration in animal models and clinical trials [31,32,33]. The mechanisms underlying stem cell therapy have largely been attributed to the paracrine actions of stem cells that are independent of their differentiation capability, as transdifferentiation is extremely rare under physiological conditions. This recognition stemmed from studies that showed that the efficiencies of myocardial recruitment and engraftment after local or systemic stem cell transplantation were typically too low to account for functional improvement [34,35,36]. It was firstly found that paracrine effects rather than transdifferentiation contribute to the functional benefits derived from MSCs in heart disease mouse models. Although it was found that transplanted MSCs derived from the bone marrow (BM-MSCs) did not undergo overt cardiomyogenic differentiation after direct injection into injured adult mouse hearts, they did improve cardiac function [37]. Subsequently, it was proposed that the production and secretion of cytoprotective factors accounts for stem cell actions in tissue protection and repair. Modified rat BM-MSCs overexpressing the survival gene Akt1 (Akt-MSCs) were shown to prevent ventricular remodeling and restore cardiac function in less than 72 h through protection of the ischemic myocardium by paracrine mediators released in situ by the BM-MSCs [19]. Furthermore, it was reported by the same group that conditioned medium (CM) from hypoxic Akt-MSCs markedly inhibited hypoxia-induced apoptosis and triggered vigorous spontaneous contraction in adult rat cardiomyocytes in vitro, and significantly limited infarct size and improved ventricular function in vivo. This finding was supported by the results for several genes, such as vascular endothelial growth factor (VEGF), hepatocyte growth factor (HGF), and thymosin b4 (TB4), which exhibited obviously upregulated expression in the Akt-MSCs, supporting the paracrine hypothesis [38]. Similarly, it was demonstrated that intravenous human MSCs (hMSCs) reduced the acute inflammatory response and infarct size and improved cardiac function in mouse myocardial infarction models, although a major population of the injected hMSCs were found to be trapped in the lungs [39]. Noninvasive intramuscular administration of BM-MSCs and MSC-CM has been reported to significantly improve ventricular function through trophic factors in a hamster heart failure model [40]. Other studies compared EV-rich fractions to CM and further indicated that EVs were responsible for the therapeutic benefits of MSC-CM [41,42]. Paracrine function has not only emerged as a mode of action of MSCs but has also inspired promising clinical applications of cytokines in regeneration and immunology. The use of MSC-secretome has advantages over the implantation of the MSCs themselves: components can be bio-modified and scaled to specific dosages, and can be stored and transported stably due to their non-living nature. However, since the components and therapeutic potency of the secretome can be influenced by cell sources, pre-conditions, isolation methods and storage conditions, there is an urgent need for standardizing the bioprocessing parameters.

## 3. MSC-Mediated Immunoregulation of Immune Cells via Paracrine Actions In Vitro

**Figure 1:**
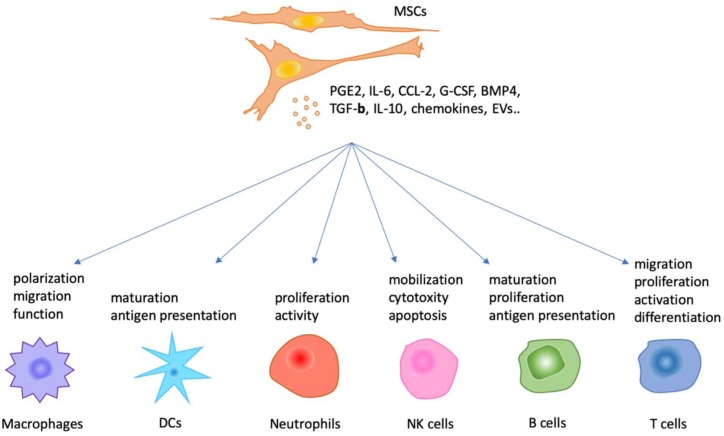
The regulatory function of mesenchymal stromal/stem cells (MSCs) via paracrine on immune cells.

It was first reported that MSCs play a critical role in the regulation of immune cells by Juneja et al. in 1986, and their study found that MSCs interacted with a monoclonal human B-lymphoblastoid cell line (UTMB-460) and participated in the establishment of the UTMB-460 cell line [43]. Subsequently, the immunoregulatory function of MSCs was gradually investigated in various studies. However, the full elucidation of mechanisms remains a matter for debate and exploration. Generally, it is hypothesized that transdifferentiation, cell–cell contact and fusion, paracrine effects, extracellular microvesicles (EVs) and mitochondrial transfer are involved in the immunomodulatory roles of MSCs [10,38,44,45,46]. Based on the observation that BM-MSCs strongly suppress T lymphocyte proliferation due to the production of soluble factors, emerging evidence suggests that the secretion of bioactive factors, including chemokines and cytokines, contributes to the broad effects of MSCs on the cells of the innate and adaptive immune systems [47] (Figure 1).

### 3.1. MSCs and Innate Immunity

Macrophages are effector cells of the innate immune system that are crucially involved in the clearance of pathogens in the initiation and resolution of immune responses [48,49,50]. MSCs affect the maturation, migration, polarization and function of macrophages by releasing factors, therefore affecting the strength and duration of immune responses and mediating tissue injury repair. It has been reported that BM-MSCs activated by lipopolysaccharide (LPS) or tumor necrosis factor-a (TNF-a) reprogram macrophages to increase the production of interleukin-10 (IL-10) by secreting prostaglandin E2 (PGE2) and attenuate sepsis and improve survival in mouse sepsis models [51]. Tumor-resident MSCs release a large amount of various chemokines, including CCL-2, CCL-7 and CCL-12, thereby enhancing the recruitment of monocytes expressing CCR2 into tumor sites and increasing the number of macrophages and growth of tumors [52]. In addition, transplanted mouse MSCs recruit M2 macrophages in a stromal cell-derived factor 1 (SDF-1)-dependent manner, which in turn promotes beta cell regeneration through the Wnt/b-catenin pathway in diabetic mouse models [53]. Human placental MSCs have been shown to polarize macrophages from an inflammatory M1 phenotype into an anti-inflammatory M2 phenotype via glucocorticoid receptor (GR) and progesterone receptor (PR) [54]. Overall, the preferential shift in macrophage phenotype from M1 to M2 mediated by MSCs may be closely related to immunoregulation and inflammatory diseases.

Dendritic cells (DCs) are the main antigen-presenting cells in the mammalian immune system. MSC supernatants inhibit CD83 expression, decrease the production of IL-12 and interfere with endocytosis during DC maturation [55]. Additionally, MSCs block the differentiation of CD14+ CD1a- precursors into dermal/interstitial DCs without affecting the generation of CD1a+ Langerhans cells. Consistent results have shown that MSCs also completely prevent the generation of immature DCs from monocytes via IL-6, M-CSF or other soluble factors [56]. A study also reported that MSCs inhibited the differentiation of DCs from bone marrow progenitors in part through the secretion of IL-6 [57]. IL-10 is a common immunosuppressive cytokine, and downstream signaling via the JAK-STAT pathway has been shown to be involved in DC differentiation and maturation. It has also been reported that MSCs inhibit the maturation of DCs through the stimulation of IL-10 secretion and the JAK1/STAT3 signaling pathway. In addition to IL-10, TNF-a-stimulating gene-6 (TSG-6) secreted by MSCs has been demonstrated to suppress MAPK and NF-kB signaling activation during the maturation of immature DCs into mature DCs induced by LPS [58]. These results suggest that MSCs maintain DCs in an immature or semimature suppressor phenotype. Intriguingly, it has been shown that in addition to the aspect of maturation, DC migratory abilities in response to CCL19 are also prevented by MSCs, thus interfering with DC antigen presentation [59,60]. Taken together, these findings indicate that MSCs disrupt the three major functions of DCs, namely, the upregulation of antigen presentation and costimulatory molecule expression, the ability to present defined antigens, and the capacity to migrate.

Neutrophils have emerged as short-lived effector cells of the innate immune system and play primary roles in the activation, orientation, and expression of adaptive immune responses [61]. When cocultured with MSCs, neutrophils are protected from apoptosis via IL-6, which is produced by the MSCs and involved in the STAT3 signaling pathway [62]. CCR2 is a chemokine receptor that is abundantly expressed on neutrophils, and TNF-a-activated MSCs secrete large amounts of CXCR2 ligands, such as CXCL1, CXCL2 and CXCL8. It has been demonstrated that MSCs recruit neutrophils through these CXCR2 ligands and that the recruited neutrophils in turn enhance tumor metastasis [63]. Furthermore, LPS-activated MSCs have been shown to augment the antimicrobial effects of neutrophils by releasing IL-8 and macrophage migration inhibitory factor (MIF) [64]. In addition to affecting the mobilization and infiltration of neutrophils, MSCs have been found to suppress unstrained neutrophil activation via increased production of superoxide dismutase (SOD3), thus attenuating neutrophil-mediated tissue damage [65]. This discovery provides insight into the application of MSC-mediated protective functions via a paracrine mechanism.

Natural killer (NK) cells are the major effector cells of the innate immune system and are endowed with the capability to kill virally infected, stressed or cancerous cells [66]. MSCs can inhibit the IL-2-induced proliferation of inactivated NK cells; moreover, MSCs are lysed by activated NK cells. IFN-y-treated MSCs can prevent the NK-mediated cytolytic effects on MSCs by increasing the expression of HLA class I on MSCs [67]. A further investigation demonstrated that MSCs altered the phenotype of NK cells and suppressed proliferation and cytokine secretion partly by secreting soluble factors, including transforming growth factor-b1 (TGF-b1) and PGE2 [68]. It was shown that indoleamine 2,3-dioxygenase (IDO) and PGE2 exert a synergistic effect on NK cells to help mediate the inhibition induced by MSCs [69]. In addition, HLA-G5 released by MSCs was reported to contribute to the immunosuppressive properties of MSCs and to inhibit NK cell-mediated cytolysis [70]. In fact, MSCs do not simply play a suppressive role in the regulation of NK cells. It has been revealed that MSCs enhance the ability of NK cells to produce and secrete IFN-y, an effect in part dependent on the soluble factors derived from MSCs as shown by the observation that conditioned medium from MSCs upregulates the expression level of IL-12b1 on the NK cell surface and the phosphorylation of STAT4 in NK cells [71]. It has also been reported that MSCs derived from Wharton’s jelly significantly increase the expansion of NK cells isolated from umbilical cord blood in the presence of IL-2, IL-15, IL-3, and FLT-3L [72]. In conclusion, although the mechanisms underlying the interaction between MSCs and NK cells remain unknown and are still under investigation, it can be suggested that paracrine effects are involved in the immunoregulation, at least in part, according to several studies.

### 3.2. MSCs and Adaptive Immunity

T cells are viewed as the primary cellular effectors of the adaptive immune system and play critical roles in antigen specificity and memory-associated cognate immunity [73,74]. When cocultured with MSCs, peripheral blood mononuclear cells (PBMCs) exhibit suppressed proliferation even without cell–cell contact, suggesting that the suppressive effects of MSCs are mediated through the release of soluble bioactive factors. A study demonstrated that HGF, IL-10, and TGF-b1 were involved in the immunosuppression of MSCs at varying concentrations in vitro. The proinflammatory cytokine IFN-y increased HGF and TGF-b1 levels, induced the expression of IDO, and contributed to MSC-mediated allosuppression [75,76]. A previous study revealed that the proliferation rate of PBMCs was inhibited 50% to 60% by human MSCs releasing suppressive cytokines, including IL-10. Furthermore, PGE2 was shown to be responsible for many of the human MSC-mediated immunomodulatory effects observed in vitro [77]. Mechanically, Toll-like receptors (TLRs) expressed by MSCs, particularly TLR3 and TLR4, function in inducing the activation of NF-kB and the production of cytokines, such as IL-6, and chemokines, such as CXCL10 and IL-8. Therefore, TLR3 or TLR4 ligation on MSCs participates in their immunosuppressive effect on T lymphocyte proliferation [78]. Consistently, it has been reported that TLRs expressed on MSCs affect the expression of cytokines including TNF-a, IL-12, and IL-1b, which has been demonstrated to be involved in DC activation and cytotoxic T cell activity [79]. In contrast, TLR2 does not affect the immunosuppressive effect exerted by murine MSCs on T cell proliferation [80]. One explanation may be that human and mouse models are different. Another recent study demonstrated that MSCs exerted different modulatory effects via a paracrine mechanism on various types of T cell subpopulations. MSC-secreted PGE2 and TGF-b1 were shown to induce CD4^+^CD25^+^FoxP3^+^ T cells [81,82]. Similarly, HLA-G5 secreted by MSCs contributes to the suppression of allogeneic T cell proliferation and CD4^+^CD25^high^FoxP3^+^ regulatory T cell (Treg) expansion [70]. It has been found that MSCs inhibit CD4 Th17 cell activation via the release of CCL2 mediated by suppressing the STAT3 signaling pathway in encephalomyelitis mouse models [83]. In addition, MSCs inhibit Th17 cell but not Treg differentiation partly through the secretion of PGE2 and IDO [84]. In addition to their direct effects on T cells, MSC-derived paracrine factors also influence T cells by regulating innate immune cells, including macrophages and DCs. It is required for T cell activation that costimulatory ligands interact with TCR on T cells. Thus, MSC-produced soluble factors can affect the expression of costimulatory ligands by APCs (antigen-presenting cells), thereby modulating T cells. For instance, MSCs affect the polarization of macrophages, thus controlling the differentiation of T cells and ultimately exerting an immunomodulatory function [85]. Collectively, the paracrine factors of MSCs convey regulatory messages to T cells through direct and/or indirect mechanisms, thereby participating in the adaptive immune system.

B cells are another major cell type involved in adaptive immune responses, acting as antigen presenters and antibody producers [86,87]. They provide costimulatory signals, secrete cytokines, affect lymphoid tissue structure and organize the splenic architecture, resulting in influences on other immune cells [88,89]. Through cell–cell interactions, MSCs facilitate AKT phosphorylation and inhibit caspase-3-mediated apoptosis in peripheral CD19+ B cells dependent on the increased expression of vascular endothelial growth factor (VEGF) [90]. Aside from direct contact, MSCs derived from adipose tissue also promote the chemotaxis and motility of B cells through the secretion of chemotactic factors. However, B cell-related chemokines produced by MSCs, including PGE2, CXCL8, CXCL10 or combinations, are not responsible for B cell chemotaxis [91]. It has been reported that MSCs inhibit B cell proliferation via cell cycle arrest in the G0/G1 phase mainly mediated by the release of soluble factors. Furthermore, MSCs downregulate the production of immunoglobulins such as IgM, IgG and IgA and decrease the expression of CXCR4, CXCR5 and CXCR7 in B cells. However, MSCs do not affect the expression of molecules involved in antigen presentation by activated B cells [13]. Similarly, it has been demonstrated that MSCs arrest the cell cycle of B cells in the G0/G1 phase. In addition, MSCs block B cell differentiation and modify the activation pattern of extracellular responses and the p38 mitogen-activated protein kinase pathways in B cells [92]. Compared to normal BM-MSCs, BM-MSCs from lupus model mice and systemic lupus erythematosus (SLE) patients show a functional defect resulting in normal B cell inhibition generally due to a reduction in CCL12 expression [93]. Interestingly, recent studies revealed that MSCs could also have a supportive influence on B cells. When cocultured with human umbilical cord-derived MSCs (UC-MSCs), B cells exhibited significant increases in proliferation and terminal differentiation marked by the expression of CD138. In addition, the UC-MSCs promoted immunoglobulin production in the B cells. The immunomodulatory effect of the UC-MSCs on the B cells was partly mediated by PGE2 but not by IL-6 [94]. Granulocyte colony-stimulating factor (G-SCF) suppresses the production of B cell trophic factors (CXCL12, IL-6, IL-7 and insulin-like growth factor-1) secreted by BM-MSCs, leading to a shift from lymphopoiesis to myelopoiesis [95]. The controversial effects of the paracrine activity of MSCs on B cells lack full understanding and merit further investigation.

### 3.3. Roles of Extracellular Vesicles

It is widely believed that the immunomodulatory function of MSCs is largely mediated by paracrine signals. Several recent studies have indicated that the regulatory effects are also partially supported by secreted extracellular vesicles (EVs). Moreover, MSC-secreted EVs (MSC-EVs) are increasingly recognized as key paracrine factors in addition to soluble factors. MSC-EVs, including microvesicles and exosomes, are a heterogeneous population of lipid membrane-encapsulated nanoparticles carrying various biomolecules, such as RNAs (mRNAs and miRNAs) and proteins (membrane receptors, enzymes, cytokines and growth factors) [96]. They play critical roles in cell–cell interactions via local or distant transfer of their bioactive cargoes from parental cells to recipient cells [97,98]. Since EVs inherit content from parental MSCs, they possess immunoregulatory characteristics or, in other words, MSCs display immunomodulation dependent on the release of EVs. It has been reported that MSC-EVs induce the M2 macrophage phenotype through mitochondrial transfer dependent on macrophage oxidative phosphorylation in acute respiratory distress syndrome mouse models [99]. Furthermore, the M2 macrophages educated by MSC-EVs promoted wound healing in the injured tendons in a mouse Achilles tendon rupture model [100]. It was similarly observed that MSC-EVs augmented an anti-inflammatory M2 macrophages and resulted in alleviation of hyperoxia-induced bronchopulmonary dysplasia (BPD), improvement of lung function, decrease in fibrosis and pulmonary vascular remodeling, and amelioration of pulmonary hypertension [101]. MSC-EVs accelerated tissue repair as well in cardiotoxin-induced skeletal muscle injury model through inducing macrophage M2-like phenotype polarization [102]. In addition to inducing phenotypic changes, MSC-EVs downregulate the levels of inflammatory cytokines (IL-22 and IL-23) and substantially increase the expression of the anti-inflammatory molecule PGE2 [103]. Additionally, it has been demonstrated that MSC-EVs attenuate antigen uptake by immature DCs and reduce DC maturation with decreased expression of CD83 and production of IL-12. Subsequently, T cells stimulated with MSC-EV-treated DCs exhibit reduced secretion of IFN-y and IL-6 [104]. A group showed that direct treatment with MSC-EVs induced T cell apoptosis without significantly suppressing cell proliferation; however, MSC-EVs strongly induced Treg proliferation and increased the expression of the anti-inflammatory cytokine IL-10 [105]. The same group also investigated the immunosuppressive effects of MSC-EVs on B cells. It was found that MSC-EVs could reproduce the inhibition of B cell proliferation and differentiation in a CpG-stimulated peripheral blood mononuclear cell coculture system in a dose-dependent manner [106]. In addition, several studies have reported that MSC-EVs induce Treg development in vivo in disease models. For instance, EVs derived from human embryonic stem cell-MSCs could induce the generation of Tregs in allogeneic skin graft models [107]. EVs from BM-MSCs suppress the immune reaction by inhibiting PBMC proliferation and enhancing Treg function, hence improving islet transplantation [108]. MSC-EVs increase the number of Tregs and levels of TGFb and HGF involved in liver regeneration in a concanavalin A-induced liver injury model [109]. Moreover, exosomes have been compared to microvesicles with variable results. Only the exosome-rich fraction induced an improvement of renal function and morphology in an acute kidney injury model, whereas the best formulation to reduce radiation damage to BM-MSCs included both types of EVs [110,111]. Compared to MSCs, cell-free EVs have fewer concerns regarding immunogenicity, tumorigenicity, storage and handling procedures, and embolism formation after administration. In particular, it is relatively easy to modify EVs to improve the effective contents and surface availability to enhance therapeutic benefits. Based on these advantages, MSC-EVs hold promising potential as novel treatments that represent an alternative to stem cell therapy.

## 4. Clinical Application of the Immunomodulation Mediated by MSCs

Further investigation and understanding of the immunomodulatory effects of MSCs have provided novel insights into the treatment of immune-mediated diseases. A variety of immune disorder diseases, including graft-versus-host disease (GVHD), Crohn’s disease, multiple sclerosis (MS), SLE and diabetes, have entered clinical trials of MSC therapies (Table 1).

### 4.1. Graft-Versus-Host Disease (GVHD)

GVHD continues to be the main barrier that limits the wider application of allogeneic hematopoietic cell transplantation (HCT) [127]. The first successful case of MSC treatment for GVHD reported BM-MSCs transplanted into a patient with severe grade IV acute GVHD in the gut and liver [112]. The patient received two rounds of transplantation, and patient lymphocyte proliferation was inhibited by 90%. This clinical trial encouraged prospective studies with MSCs for GVHD treatment [113,114]. Another clinical study enrolled 75 patients and reported that the overall response rate at day +28 was 61.3%, and this response was correlated with significantly improved survival at day +100 after MSC infusion [115]. However, some studies have shown inconsistent results regarding efficiency and safety. In one study, only two patients (15%) responded, and another 11 patients required further escalation of immunosuppressive therapy when the median dose of MSC cells was 2 × 10^6^/kg (range 1–5). Although a follow-up study supported the efficacy of MSCs in the treatment of steroid-refractory acute GVHD, the response to MSC transfusion was lower than that in previous cases [116]. Similarly, in a study of 11 patients who received intravenous MSCs for GVHD at a median dose of 1.2 × 10^6^/kg (range 0.7–3.7), only four patients achieved a complete response initially, while four patients presented GVHD recurrence between two and five months, and two patients developed chronic limited GVHD [117]. The dose of MSCs used in current clinical trials ranges from 1 × 10^6^ to 2 × 10^8^ cells/kg, and the number of administrations varies from one to eight [128]. The inconsistent outcomes might result from variation in the translation from preclinical studies to clinical application, including the homogeneity of the MSCs, the optimal dose and frequency of administration, and the complicated physical conditions of the patients.

### 4.2. Crohn’s Disease

Crohn’s disease (CD) is a chronic inflammatory bowel disease characterized by inflammation in the digestive or gastrointestinal tract. A study showed that a local injection of autologous MSCs increased mucosal and circulating Treg frequencies in patients with CD [118]. Subsequently, the continuation of the study reported the outcome of a five-year follow-up study of the phase 2 trial. Disease remission was observed 12 months after MSC infusion, and the mean CD activity index (CDAI) score increased significantly, followed by a gradual decrease with eventual remission achieved at the end of the five-year follow-up period. The probability of fistula relapse-free survival was 88% at one year but decreased to 37% during the following four years [129]. A phase 2 study included 16 patients who received intravenous infusions of allogeneic MSCs (2 × 10^6^/kg) weekly for four weeks and showed decreased CDAI scores after each MSC infusion. Twelve of 15 patients had a clinical response, and eight achieved clinical remission [119]. A phase 1 trial also proved the feasibility and safety of autologous BM-MSC therapy for CD. Twelve patients received a single MSC intravenous injection of 2, 5 or 10 × 10^6^ cells/kg, and all patients tolerated the infusion well [120].

### 4.3. Multiple Sclerosis

Multiple sclerosis (MS) is a demyelinating and chronic inflammatory disease of the central nervous system [130]. It was first reported that 10 patients with progressive-type MS received autologous MSC transplantation in 2007. After MSC treatment, they did not experience any major adverse events, and during 13 to 26 months of follow up, the expended disability status scale (EDSS) score of one patient improved from 5 to 2.5, the scores of five patients increased from 0.5 to 2.5, and the scores of four patients showed no change. This research confirmed the feasibility of using autologous MSCs for the treatment of MS patients [121]. Subsequently, a pilot study investigated the safety and therapeutic benefits of autologous BM-MSCs in primary progressive MS patients. MSCs were infused into the subarachnoid space at the C1-C2 and L2-L3 disc space levels. Over the next six months, EDSS scores improved by 0.5–1.0 in five of seven patients, remained unchanged in one patient, and worsened by 0.5 in one patient. The overall attrition rate was 30%, and brain MRI showed no new or gadolinium-enhancing lesions in any patient [122]. These results reconfirmed the efficacy of MSC treatment for MS. In addition, a phase 2 study reported that MSC infusion produced improvements in patients with secondary progressive disease, particularly in measures of visual function, physiology, and structure, without significant adverse events [123]. The precise mechanism of MSC-mediated immunomodulation in MS is not known and requires further exploration.

### 4.4. Other Immune-Related Diseases

Since MSCs were found to be able to differentiate into insulin-producing cells, MSC therapy has emerged as a promising approach for diabetes. It was reported that MSC injection via liver puncture in two patients with type 1 diabetes reduced the expression levels of islet cell antibody (ICA), glutamic acid decarboxylase (GAD) and anti-insulin antibodies, and increased the level of C-peptide [124]. In addition, systemic MSC treatment at a mean dose of 2.75 × 10^6^ cells/kg via intravenous administration could improve the C-peptide response in patients with type 1 diabetes [125]. Based on the therapeutic effects of MSCs on atopic dermatitis (AD) in preclinical studies, the safety and efficacy of MSCs in clinical application has been investigated. Thirty-four patients with moderate-to-severe AD received subcutaneous injection of hUCB-MSCs at a dose of 2.5 × 10^7^ or 5 × 10^7^/kg and subsequently rapidly improved without adverse events. The case report suggested that efficacy was dependent on the dose and frequency of injection of hUCB-MSCs [126]. Actually, it is difficult to assess the benefits of MSC therapy by comparing the results of published studies because of the small number of patients involved in most clinical trials and various different approaches in regard to MSC sources, preparation, administration and efficacy, and safety tests (Table 2). In addition, the implied possible complications of MSC cell therapy are associated with the immunosuppressive properties of MSCs due to the reduction in the immunosurveillance of host neoantigens and foreign pathogens or viruses [131]. In addition, autologous MSCs may induce tumors by promoting tumor cell growth, whereas allogeneic MSCs derived from donors may increase infectious risk. Secreted factors or conditioned medium may offer advantages over cell-based therapy because of the more specific contents and definite signaling pathways involved, thereby producing the expected clinical outcome. Thus, the paracrine function of MSCs provides the possibility of applying a specific factor alone or in combination as a cocktail therapy for treatment.

## 5. Conclusions

MSCs have emerged as a promising candidate for therapeutic application due to their multidifferentiation potential and immunomodulatory properties. In addition to performing functions via cell–cell contact, MSCs exert immunoregulatory functions through the combined reactions of chemokines, cytokines, and extracellular vesicles secreted by MSCs, as well as the microenvironment and inflammatory stimuli. Surprisingly, although the immunomodulatory capacities of MSCs have only recently been confirmed, MSC therapies are of great interest and widely explored, and approval has been obtained for clinical trials treating inflammatory or autoimmune diseases in the United States, Europe, and China. Accumulating clinical studies have demonstrated the excellent therapeutic benefits of MSC therapy for the treatment of several immune diseases due to the immunomodulatory function of MSCs. However, the risks of MSC therapy include immunosuppression, ectopic differentiation and tumor growth promotion. Paracrine-based, cell-free therapy holds great promise as a controllable, manageable, and feasible alternative. We believe that further investigation of the molecular mechanisms and signaling networks that regulate paracrine activities will promote the clinical application from bench to bedside in the near future.

## Figures and Tables

**Table 1:** Summary of the clinical application of MSCs.

Disease	Sample Size	Study Period	Origin	Dosage	Injection	Reference
aGVHD	1	1 year	allogeneic BM MSCs	1, 2 × 10^6^/kg	i.v.	[112]
aGVHD	55	60 months	allogeneic BM MSCs	1.4 × 10^6^/kg	i.v.	[113]
aGVHD	12	427–1111 days	allogeneic BM MSCs	8 × 10^6^/kg 2 × 10^6^/kg	i.v.	[114]
aGVHD	75	2–1639 days	allogeneic BM MSCs	2 × 10^6^/kg	i.v.	[115]
aGVHD	13	55–692 days	allogeneic BM MSCs	0.9 × 10^6^/kg	i.v.	[116]
GVHD	11	4–18 months	allogeneic BM MSCs	1.2 × 10^6^/kg	i.v.	[117]
CD	12	1 year	autogenous BM MSCs	2 × 10^7^/kg	Lumen and the walls of the tracks	[118]
CD	16	6 weeks	allogeneic MSCs	2 × 10^6^/kg	i.v.	[119]
CD	12	2 years	human placenta-MSCs	2, 5, 10 × 10^6^/kg	i.v.	[120]
MS	10	13–26 months	autogenous BM MSCs	8.73 × 10^6^/person	Intrathecally	[121]
MS	10	1 year	autogenous BM MSCs	3–5 × 10^7^/person	The subarachnoid space	[122]
MS	10	20 months	autogenous BM MSCs	1.6 × 10^6^/kg	i.v.	[123]
T1D	2	1 year	autogenous BM MSCs	180 × 10^6^/kg	Liver puncture	[124]
T1D	20	1 year	autogenous BM MSCs	2.75 × 10^6^/kg	i.v.	[125]
AD	34	1, 3 months	human UCB MSCs	2.5, 5 × 10^7^/kg	Subcutaneously	[126]

aGVHD, acute graft versus host disease; CD, Crohn’s disease; MS: Multiple sclerosis; T1D: type 1 diabetes; AD: atopic dermatitis; i.v.: intravenously; BM MSCs: MSCs derived from bone marrow; UCB MSCs: MSCs derived from umbilical cord blood.

**Table 2:** The challenges in clinical application of MSC therapy.

Problems	Detailed Questions
Origin of MSCs	Currently commonly used: bone marrow, adipose tissue, umbilical cord
	Other choices remain to be explored: dental pulp, thymus, gingiva, saphenous vein, fetal tissues
Ex-vivo preparation	Age of donors
	Ex-vivo culture conditions
	Specific genetic modification
Protocols of injection	Cell dose and frequency
	Transfusion way
	Combined with chemotherapy
Assessment	Efficacy test
	Safety test
	Follow-up study
